# Semi-field experiments reveal contrasted predation and movement patterns of aquatic macroinvertebrate predators of *Anopheles gambiae* larvae

**DOI:** 10.1186/s12936-025-05242-8

**Published:** 2025-01-09

**Authors:** Hudson Onen, Emmanuel W. Kaindoa, Joel Nkya, Alex Limwagu, Martha A. Kaddumukasa, Fredros O. Okumu, Jonathan K. Kayondo, Anne M. Akol, Frédéric Tripet

**Affiliations:** 1 Department of Zoology, Entomology and Fisheries Sciences, College of Natural Sciences, School of Biosciences, Makerere University, P.O. Box 7062 Kampala, Uganda; 2 Department of Entomology, Uganda Virus Research Institute, Plot 51/59 Nakiwogo Road, P.O. Box 49, Entebbe, Uganda; 3 Environmental Health and Ecological Science Department, Ifakara Health Institute, P.O. Box 53, Ifakara, Tanzania; 4 Faculty of Science, Biological Sciences, Kyambogo University, P.O. Box 1, Kampala, Uganda; 5 Swiss Tropical and Public Health Institute, Kreuzstrasse 2, 4123 Allschwil, Switzerland; 6 University of Basel, Petersplatz 1, 4001 Basel, Switzerland

**Keywords:** Macroinvertebrate, Predation, *Anopheles gambiae*, Semi-field, Larval aquatic habitats

## Abstract

**Background:**

Members of the *Anopheles gambiae* complex are major malaria vectors in sub-Saharan Africa. Their larval stages inhabit a variety of aquatic habitats in which, under natural circumstances, they are preyed upon by different taxa of aquatic macroinvertebrate predators. Understanding the potential impact of predators on malaria vector larval population dynamics is important for enabling integrated local mosquito control programmes with a stronger emphasis on biocontrol approaches. This study experimentally evaluated the predation efficacy and foraging strategy of three common aquatic macroinvertebrate predators of *An. gambiae*, diving beetles (Coleoptera), backswimmers (Hemiptera), and dragonfly nymphs (Odonata) in a semi-field system in South-Eastern Tanzania.

**Methods:**

An array of alternating small and large basins used as aquatic habitats was created in two compartments of a semi-field system and filled with well water. Field-collected adult diving beetles, backswimmers or dragonfly nymphs were randomly assigned to these habitats and *Anopheles arabiensis* larvae were added as prey in half of the habitats. The number of mosquito larvae consumed, predator mobility across habitats and mortality were recorded at 24, 48 and 72 h.

**Results:**

The presence of *An. gambiae* larvae in habitats significantly increased the survival of backswimmer and dragonfly nymphs, which are not mobile. In contrast, diving beetles survived well under any initial condition by preferentially flying away from habitats without prey to nearby larger habitats with prey. The larval predation rates of predacious diving beetle, backswimmer and dragonfly nymphs were stable over time at a mean of 3.2, 7.0 and 9.6 larvae consumed each day.

**Conclusion:**

This study demonstrates that aquatic macroinvertebrate predators display adaptive foraging behaviour in response to prey presence and aquatic habitat size. It also confirms the ability of these predators to significantly reduce *An. gambiae* larval densities in aquatic habitats, thus their potential for consideration as additional biocontrol tools for mosquito population reduction.

## Background

Members of the *Anopheles gambiae* complex are the most efficient vectors of malaria in sub-Saharan Africa [1, 2]. Currently, two main strategies are used in Africa to combat malaria: rapid diagnostic tools to identify *Plasmodium*-infected patients combined with drug treatment, and vector control tools designed to reduce mosquito populations or prevent biting on human hosts [3]. The most crucial of these strategies for reducing human morbidity is vector control, which, since the last century, relied heavily on using chemical insecticides to limit populations of malaria mosquitoes [4, 5]

In the 1950s, the chemical insecticide Dichlorodiphenyltrichloroethane (DDT) was first introduced [6]. Spraying campaigns with DDT saved approximately one billion people from malaria and demonstrated that the disease could be controlled and, in some areas, eradicated [7]. Consequently, the World Health Organization (WHO) heavily promoted the use of DDT until the 1980s, when widespread mosquito resistance to DDT was reported across all major malaria vector species [8, 9]. Furthermore, alarming evidence emerged of DDT's negative effects on animal health and the environment due to its accumulation in natural food chains, prompting the WHO to encourage discontinuing its use [8].

The end of DDT campaigns caused a rebound of malaria in endemic countries, and by the 1990s, the annual death toll from malaria reached two million, mainly among children under the age of five [10]. The Roll Back Malaria (RBM) campaign was launched in 1998, taking advantage of pyrethroids, a newly discovered class of insecticides that demonstrated very low toxicity to vertebrates [11, 12]. Roll Back Malaria further relied on chemical-based interventions, such as indoor residual spraying (IRS) and novel insecticide-treated nets (ITNs) as primary vector control tools [13, 14]. In the past 20 years, these tools have been instrumental in reducing the death toll from malaria [14–16]. However, the rapid spread of insecticide resistance, combined with changes in species dynamics and the biting behaviour of most malaria vectors, is currently impeding the efficacy of ITNs and IRS [17, 18]. As a result, there is a renewed interest in developing malaria vector control approaches that emphasize integrated strategies and non-chemical tools [19].

Before the introduction of DDT in the 1940s, malaria vector control programs often relied on holistic, location-specific strategies that used a variety of larval source management approaches such as environmental management, larviciding and biocontrol [20]. Because these approaches did not rely on chemical pesticides, they allowed the natural buildup of natural predator populations that regulate mosquito populations. Unfortunately, this is no longer the case due to an over-reliance on chemical insecticides in agriculture and vector control [21]. Currently, in most African countries, rice cultivation is accompanied by intensive chemical pesticide applications targeting stem and leaf-eating Lepidoptera, Orthoptera, and Coleoptera insect species [22]. This has resulted in a rise in insecticide resistance among the malaria vectors due to the selective pressure from prolonged exposure of their juvenile stages to the chemicals in aquatic habitats [23–26]. Additionally, these chemicals have a detrimental effect on natural aquatic predators of mosquitoes, preventing them from reducing mosquito populations through density-dependent processes [27].

The use of biological organisms could successfully replace chemical control interventions in long-term integrated community-based vector control programmes [28]. Coleoptera: Dytiscidae, Odonata: Lestidae and Libellulidae, and Hemiptera: Notonectidae, are naturally occurring aquatic macroinvertebrate predators known to co-exist with and prey on *An. gambiae* larvae in wetlands including ponds, roadside ditches and temporary pools [29–31]. Populations of these natural mosquito larvae predators can be locally sustained and used against other local malaria vector species since they prey on a variety of mosquito species and other organisms in their natural habitats [32].

Predator-larvae interactions are the main mechanisms that influence mosquito larval mortality in natural settings [33]. Like other predators, aquatic macroinvertebrate predators need to search for and find prey in their habitats to feed [34]. The foraging behaviour of animals naturally varies across taxa [34, 35]. Optimal foraging theory predicts that the most important criteria guiding the behaviour of predatory organisms are the energy value of prey and foraging costs. Predators will preferentially engage in foraging behaviours that maximize energy returns in a short period [36, 37].

Understanding predators' optimal foraging behaviour is a long-standing area of interest in behavioural ecology research [34]. However, little is known about the foraging behaviour of aquatic macroinvertebrate predators of *An. gambiae* larvae. This study evaluated the predation efficacy and foraging strategy of the three most common aquatic macroinvertebrate predators identified in the natural *An. arabiensis* larval habitats in rural villages of south-eastern Tanzania through experiments in a semi-field system. The results add to our knowledge of the predation efficiencies and movements of aquatic macroinvertebrate predators across aquatic habitats, which is important for improving the design and application of larval source management approaches towards reducing malaria vector populations.

## Methods

### Mosquito collection

**Fig. 1:**
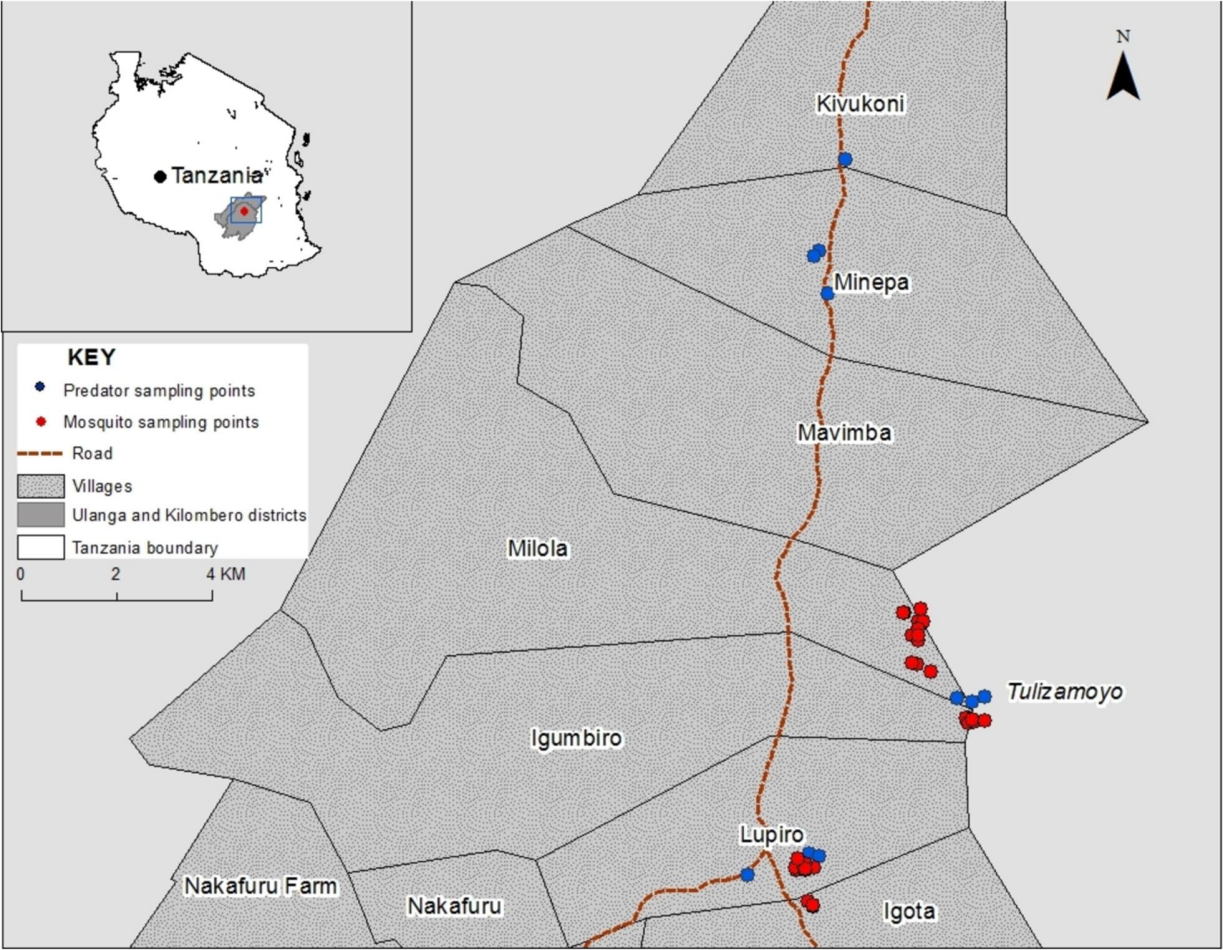
Mosquito and aquatic macroinvertebrate sampling points along the road to and in Lupiro and Tulizamoyo villages

Wild blood-fed female mosquitoes were collected using mouth aspirators between 6:00 and 7:00 h from accessible huts in Tulizamoyo (8.3545° S, 36.7055° E) and Lupiro (8.3862° S, 36.6723° E) villages in the Ulanga district between December 2021 and May 2022 (Fig. 1). Morphologically identified Anophelines were transferred into the field collection mosquito cages (15 × 15 × 15 cm) and provided with a 10% sugar solution soaked on a cotton pad placed on top of the cage's nets. The mosquitoes were transported to the vector-sphere insectary of the Ifakara Health Institute (IHI).

### Individual female mosquito egg-laying

Upon delivery to the insectary, mosquitoes were held for 72 h while being provided with a 10% sugar solution to provide energy for survival and successful egg maturation. Consequently, individually fully gravid mosquitoes were aspirated from their respective cages using mouth aspirators and transferred into each Eppendorf tube containing moist filter paper, labelled, and left overnight to lay eggs. Tubes with eggs and the mosquitoes were refrigerated at 20 °C for 15 min, and each individual mosquito was removed using forceps and transferred into a separate 1.5 mL vial containing 80% ethanol for species identification. Eggs from each tube were transferred to labelled larval trays for rearing following standard protocol [38].

### Mosquito species identification

Genomic DNA was extracted from a single mosquito leg following a standard protocol [39]. Species-specific nucleotide sequences in ribosomal (rDNA) intergenic spacers were used to distinguish *An. arabiensis* from other species of the *An. gambiae* complex [40]. A PCR reaction was conducted in a final volume of 25 µl to molecularly confirm the species identity of each specimen. The master mix for each reaction consisted of deionized water (10.3 µl), 5X buffer (2.5 µl), 10 mM dNTPs (1 µl), (Invitrogen life technology, USA), each *An. gambiae* complex (UN, ME, AR, GA and QD) primer (1 µl) (Eurofins Genomics, Germany), 50 mM MgCl_2_ (2 µl) (Invitrogen life technology, USA), and 1XTaq DNA polymerase (0.2 µl) (Platinum Taq Invitrogen, USA). Next, 3 µl of the extracted DNA was added as a template. The DNA was amplified in a thermocycler under the following conditions: one cycle holding at 95 °C for 5 min; 30 cycles of denaturation at 94 °C for 30 s; annealing at 58 °C for 30 s; extension at 72 °C for 30 s followed by one hold at 72 °C for 5 min. The PCR products were separated by gel (2.5% agarose)-electrophoresis under 0.05 M Tris–borate-EDTA buffer (Sigma-Aldrich, USA) running at 90 V for 45 min. The gel was stained with 10 mL Ethidium bromide (Sigma-Aldrich, USA) and the separated amplified fragments were visualized by illumination with short-wave ultraviolet light and fragment sizes were estimated using a 100 bp DNA ladder.

### Mosquito colony refreshment

Based on PCR results, only larvae from female mosquitoes confirmed to be *An. arabiensis* were reared to adults following standard procedures [38]. Mosquito pupae from wild-caught blood-fed females and the Ifakara insectary-reared mosquito colony were morphologically sexed under stereo microscopes. Consequently, wild-caught male and insectary-reared female pupae were placed in their respective paper cups at a ratio of 1:1 and then transferred to a single rearing cage of dimensions 35 × 35 × 35 cm with a wooden base of dimensions 37 × 37 × 37 cm. A similar procedure was followed with the wild-caught female and insectary-reared male pupae. The pupae were left for two days to allow for adults to emerge. The genetically refreshed filial one (F1) resulting from mated adults and subsequent generations was maintained following standard rearing procedures [38].

### Setting of experimental aquatic habitats in the semi-field

**Fig. 2:**
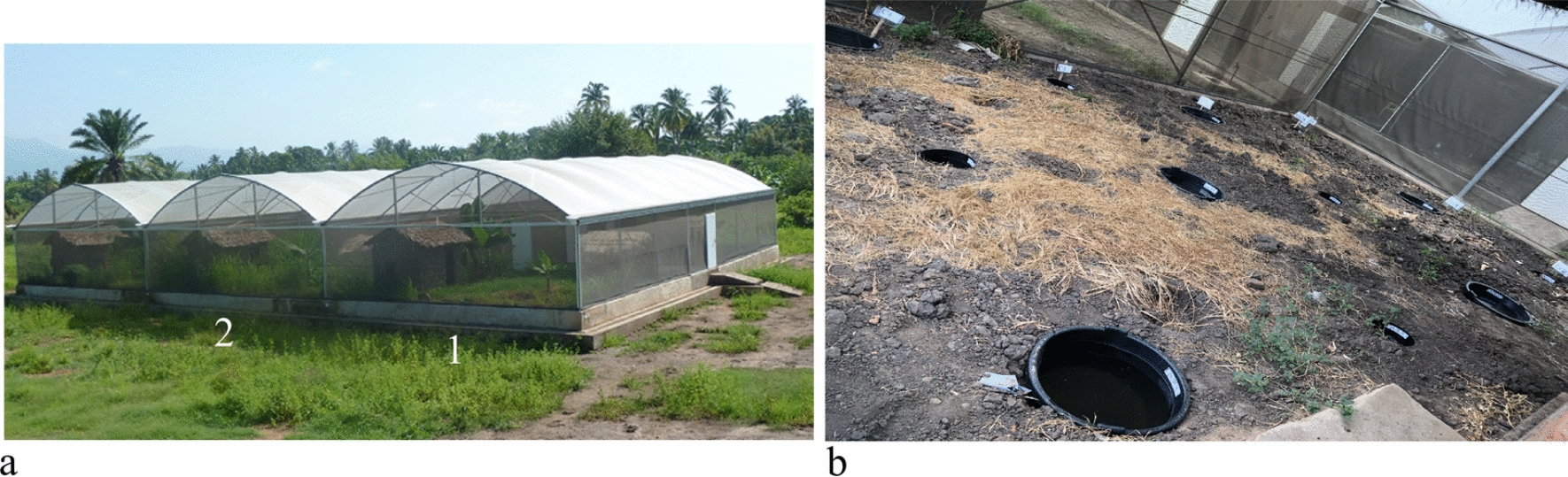
Semi field facility where compartments 1 and 2 were used for predator behaviour experiments **a** using small and large experimental aquatic habitats **b**

The semi-field system (SFS) used is a 625 m^2^ greenhouse frame with mosquito netting covering the walls and a polyethene roof mounted on a raised concrete platform. The interior is divided into six compartments with each containing a mud-walled hut and natural vegetation including banana plants (Fig. 2a). The SFS is located in Kining’na village, south-eastern Tanzania [41].

For this experiment, two compartments each measuring 8.6 × 9 m (L*W) were selected. In each compartment, six small (30 cm diameter and 20 L capacity) and six large (60 cm diameter and 40 L capacity) black-coloured basins were planted in the soil 2 m apart in a randomized Latin square design of 3 rows by 4 columns (Fig. 2b). The basins were then filled with well water available at the site and referred to as aquatic habitats.

### Sampling of aquatic macroinvertebrate predators

Before the initiation of the experiment, adult diving beetles (hereafter referred to as diving beetles), backswimmer nymphs, and dragonfly nymphs were collected using a standard 500-micron nylon mesh aquatic-D net from the semi-permanent water pools along the road to and in the villages of Lupiro and Tulizamoyo and surroundings (Fig. 1). The sampled predators were identified to family level using an identification guide [42], and their body length was measured using a ruler of 1-mm precision. Diving beetles (15–20 mm), backswimmer nymphs (4–6 mm) and dragonfly nymphs (10–12 mm) were among the most encountered predators in the sampled semi-permanent habitats. Fifteen individuals of each predator taxon were placed in a 10-L bucket filled with 5 L of clean water obtained from its sampling point. The bucket lids were covered with netting, and the collected samples were transported within 1–2 h to the experimental semi-field facility in the Kining’na field site of IHI..

### Experimental design

Eight diving beetles of approximately similar sizes were selected, and their elytra were uniquely marked with white oil paint using toothpicks. Since backswimmer and dragonfly nymphs do not fly, they were not paint-marked. Predators were then regularly alternated over the array of 12 habitats (3 rows by 4 columns) to achieve a fully balanced randomized design in relation to predator taxa. The unique marks on the elytra of the diving beetles corresponding to the habitats in which each beetle was placed was recorded. Using a 3 mL Pasteur pipette, 10 and 20 third instar larvae of the genetically refreshed *An. arabiensis* were introduced to half of the small and large habitats, respectively as prey. Habitats with and without mosquito larvae were alternated to maintain full design randomization, and they were labelled 'Fed' and 'Unfed', respectively. Each day (24, 48, and 72 h) was treated as an independent experiment. After 24 and 48 h, the mosquito larvae consumed in each habitat were replenished to restore their original numbers (10 larvae in small and 20 in large basins).

### Data collection

Mosquito larval numbers, dead and alive predators and their movements across the aquatic habitats were recorded at 24, 48, and 72 h. At each session of data collection, diving beetles that flew to other aquatic habitats were returned to the aquatic habitats where they were initially placed. On each consecutive day, the starting position of data collection in each compartment was altered. The study was repeated four times, and each repeat was considered a replicate. Between each replicate, aquatic habitats were emptied, washed, and dried for 12 h before the next experiments.

### Data analysis

All data were analysed using JMP version 14 software (SAS Institute, Inc., USA). Data were checked for deviations from normality and heterogeneity, and analyses were carried out using parametric and non-parametric approaches as required. Data from all replicates were used for analysis, and replicate effects were tested but were only reported when significant. Interactions between independent variables were tested using stepwise models, and only those significant ones were retained in the final models. Nominal logistic regression was used to determine factors influencing the movements and mortality of fed and unfed predators across habitat sizes. Generalized linear regression using Poisson distribution was used to determine the factors influencing the mean and total daily numbers of mosquito larvae eaten. Chi-square proportion likelihood tests were used to determine the most common habitat type from which moving diving beetles originated, and the distance and characteristic of their preferred destination habitats.

## Results

### Predator survival

The daily predator survival over the 3 days (72 h) in both habitats was 94.66% and did not differ significantly across taxa (Chi-square likelihood: χ2 = 0.43,* p* = 0.833). The percentage survival of dragonfly nymphs was 95.69% (95% CI 0.89–0.98), backswimmer, 94.57% (95% CI 0.88–0.99), and diving beetles 93.75% (95% CI 0.87–0.97).

**Fig. 3:**
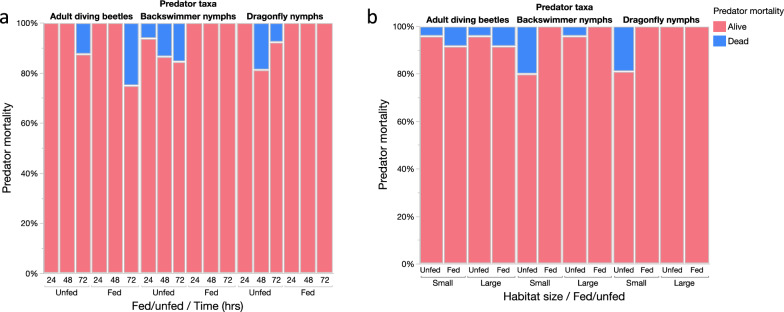
Predator’s nightly survival in relation to **a** prey availability and time (24, 48 and 72 h) and **b** prey availability and habitat size

Logistic regression analyses showed that the daily survival of diving beetles was significantly affected by the time of the experiments (Chi-square likelihood: ratio: χ2 = 14.11,* p* = 0.009) but not habitat size and prey availability in the habitats (Table 1, Fig. 3a–b). On the other hand, the survival of backswimmer nymphs was significantly affected by the presence of prey in the habitats (χ2 = 8.59,* p* = 0.003) but not habitat size and time (Table 1, Fig. 3a–b). The dragonfly nymph survival was significantly affected by habitat size and presence of prey (Chi-square likelihood: χ2 = 6.90,* p* = 0.009 in both cases) but not by time (Table 1, Fig. 3a–b).

**Table 1:** Independent stepwise nominal logistic regressions of the effect of time, prey availability and habitat size on survival of each predator taxon

Predator taxa	Parameter	df	Likelihood ratio	*p*-value
Diving beetles	Time	2	14.11	0.009
	Fed/unfed	1	0.833	0.361
	Habitat size	1	0.00	1.000
Dragonfly nymphs	Time	2	4.86	0.088
	Fed/unfed	1	6.90	0.009
	Habitat size	1	6.90	0.009
Backswimmer nymphs	Time	2	1.05	0.591
	Fed/unfed	1	8.59	0.003
	Habitat size	1	3.15	0.008

### Predator movements and habitat preference

**Fig. 4:**
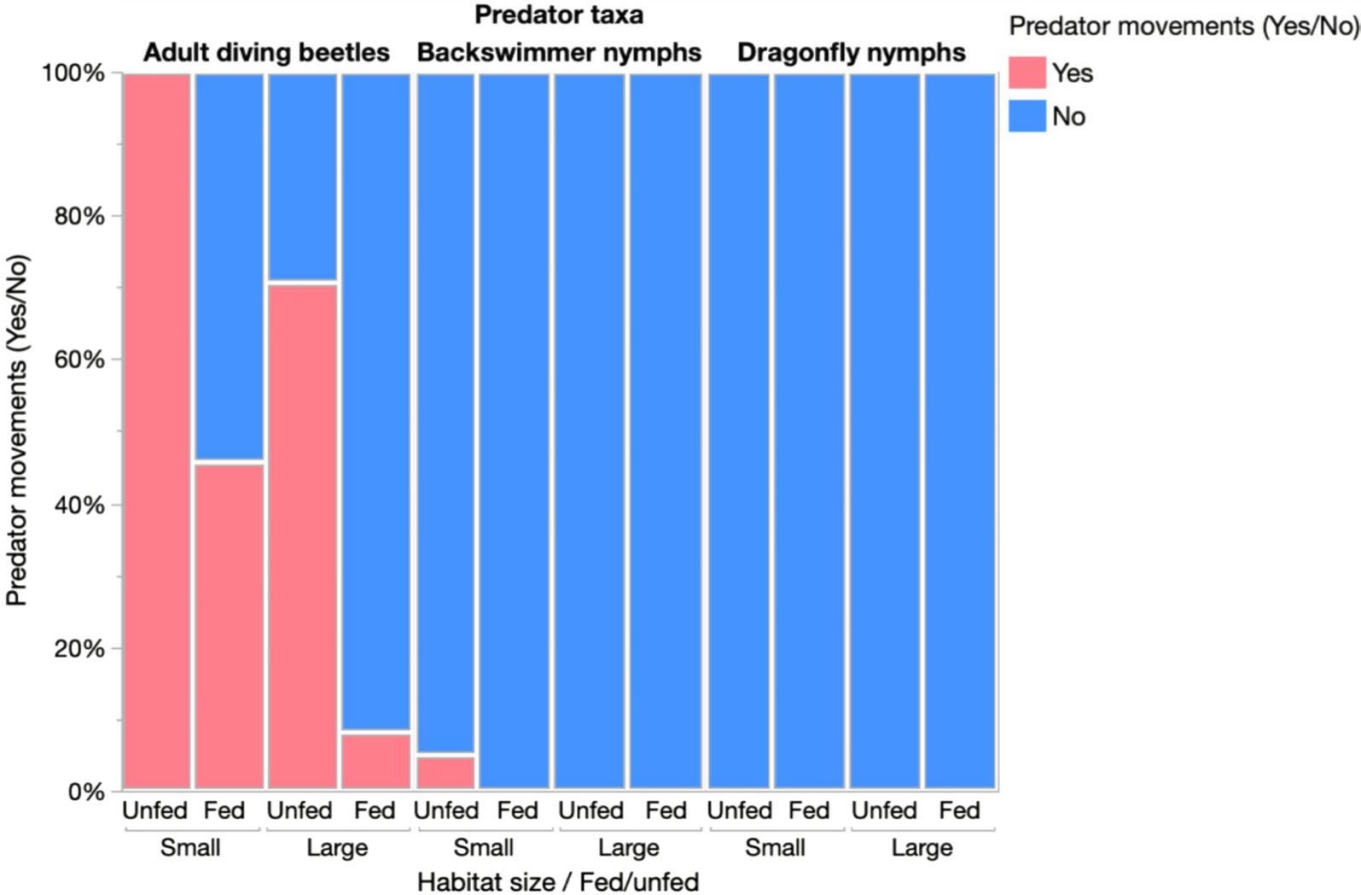
Likelihood of predator’s movement across habitats with and without prey

Overall, diving beetles recorded the highest percentage, 100% of movement from small habitats without prey compared to only 2.78% of movement from large habitats with prey (Fig. 4). A single backswimmer also left small environments devoid of prey. Dragonfly nymphs did not move from any habitat, whether it included or did not contain mosquito larvae (Fig. 4).

**Fig. 5:**
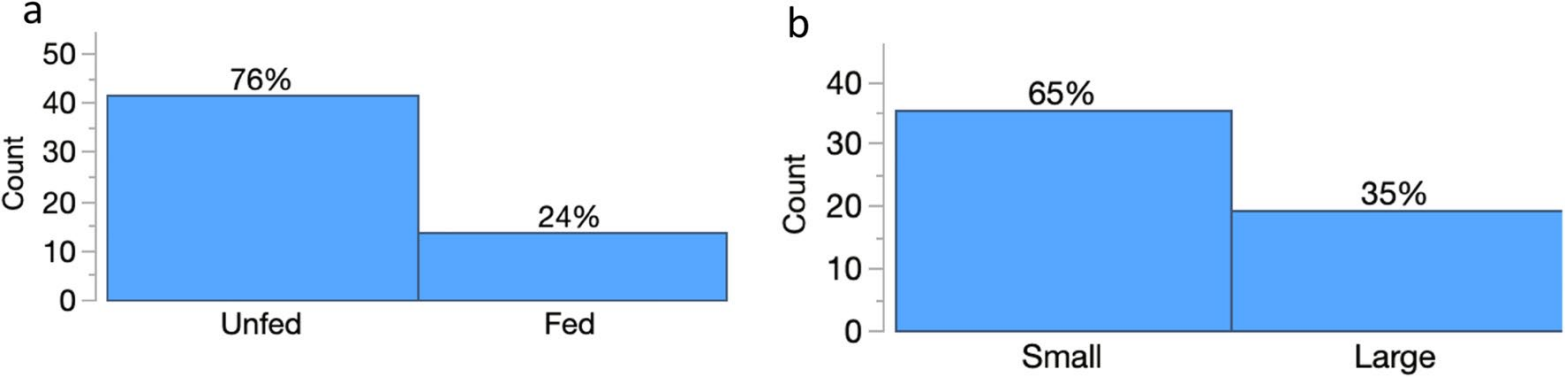
Frequency of overnight beetles’ movements from their original habitat in relation to: **a** the presence of *An. gambiae* larvae prey; and **b** habitat size

Nominal logistic regression analysis showed that beetles were significantly more likely to move when they were in a small habitat and from habitats without prey (Table 2, Figs. 5a, 5b). The likelihood of beetles’ movement did not vary significantly between subsequent days (Table 2).

**Table 2:** Logistic regression of the effect of habitat size, prey availability and time on the likelihood of movement of diving beetles between habitats overnight

Parameter	df	Likelihood ratio	*p*-value
Time	2	1.63	0.442
Fed/unfed	1	23.57	< 0.001
Habitat size	1	9.44	0.002

**Fig. 6:**
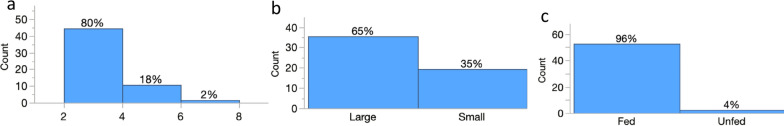
Characteristics of overnight adult diving beetle’s destined habitats **a** distance to destined habitat (m); **b**, destined habitat size; **c** and prey availability

Diving beetles that moved did so to habitats close to the habitats where they were initially placed (Fig. 6a). They significantly preferred moving to large nearby habitats (Chi-square ratio test habitat size: χ2 = 18.59,* p* < 0.001) and habitats with prey (Chi-square ratio test fed/unfed: χ2 = 43.15,* p* < 0.001) (Figs. 6a, 6b).

### Eaten mosquito larvae

**Fig. 7:**
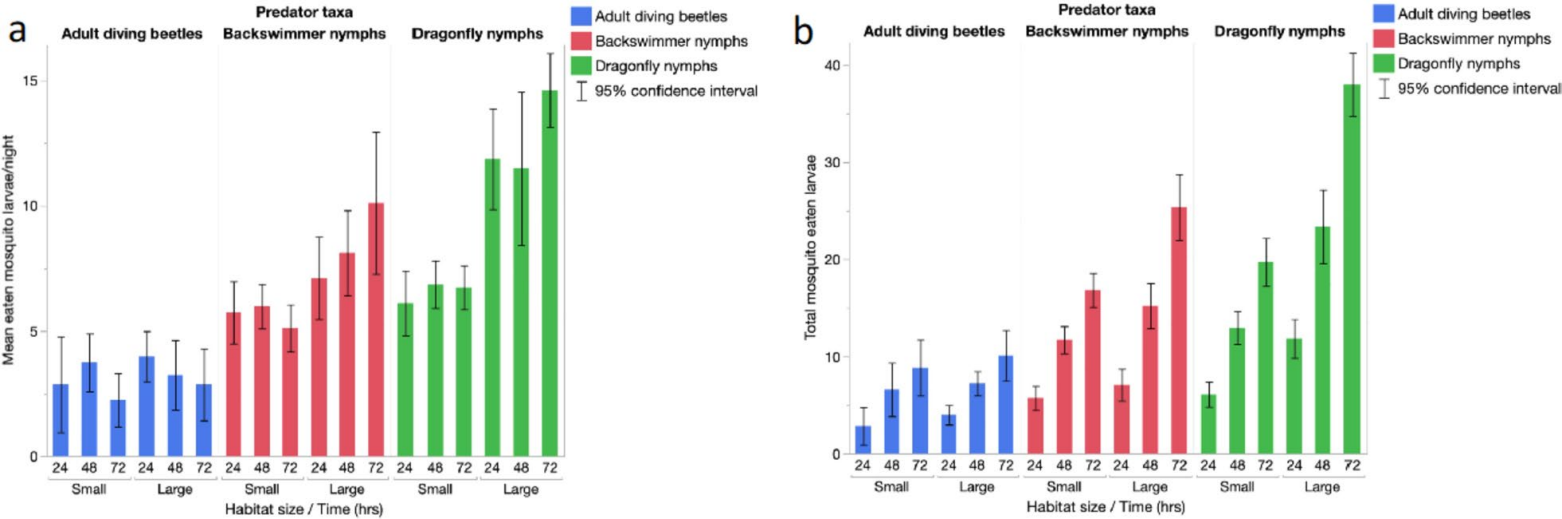
Mean **a** and total eaten **b** mosquito larvae across habitats by predator taxa at 24, 48 and 72 h

Overall, the number of mosquito larvae eaten varied significantly across predator taxa (ANOVA: *F*_143,2_ = 63.98, *p* < 0.001). Diving beetles had the lowest predation efficiency with a mean of 3.12 (95% CI 2.36–3.97) larvae consumed per day, backswimmer nymphs were second with 7.04 (95% CI 6.24–7.85) prey consumed daily and dragonfly nymphs had the highest predation efficiency of 9.63 (95% CI 8.82–10.43) (Fig. 7a). Predacious beetles consumed a total of 39.75 mosquito larvae, backswimmers nymphs, 47.75 larvae and dragonfly nymphs, 112.13 larvae over the course of the 3 day experiment in both habitats (Fig. 7b).

Generalized linear model regression confirmed the significant effect of predator taxa on predation rates. Additionally, predation rates were significantly higher in large habitats, and the effect of habitat size differed between predator taxa, as highlighted by a significant interaction between these two parameters (Table 3). There was no impact of habitat size on the number of prey that beetles consumed per day. However, both backswimmer and dragonfly nymphs consumed more larvae in the larger habitats that contained more larvae. Time (24, 48 and 72 h) did not influence predation efficiency within the timespan of the experiment (Table 3).

**Table 3:** General linear model (Poisson distribution) of the effect of habitat size, predator taxa and time (24, 48 and 72 h) on the number of mosquito larval numbers consumed overnight

Parameter	df	Chi-square (χ2)	*p*-value
Habitat size	1	29.67	< 0.001
Predator taxa	2	144.72	< 0.001
Time	2	1.62	0.445
Habitat size* predator taxa	2	8.17	0.017

## Discussion

This study provides the first insight into the adaptive foraging behaviour of common macroinvertebrate predator taxa of *An. gambiae* s.l. in sub-Saharan Africa, the diving beetles, dragonflies, and backswimmers nymphs under semi-field conditions in rural south-eastern Tanzania.

The results of this study demonstrated that dragonfly and backswimmer nymphs had higher daily survival rates than diving beetles and much higher daily survival in habitats where *An. arabiensis* larvae were available as prey. This is probably because the mosquito larval numbers were kept constant in each fed habitat by daily replenishment, ensuring a constant availability of food throughout the 72 h study. Unlike diving beetles, dragonfly and backswimmer nymphs could not move between habitats, and their survival depended entirely on the presence or absence of mosquito larvae as prey in their habitats. The only exception was a single backswimmer that underwent its final moult and became a mobile adult between the time it was collected in the field and the end of the experiment. Backswimmer and dragonfly nymphs assigned to “unfed” habitats experienced a 5.43% and 4.30% decrease in survival compared to those in “fed” habitats. Notonectids are voracious predators requiring a large number of prey to sustain their survival compared to other predator taxa [43], hence their observed reduced survival in this study. Backswimmers and dragonfly nymphs in small “unfed” habitats also had 20% and 19.05% mortality, respectively, compared to 4.18% and 0% in larger “unfed” habitats indicating that conditions in the smaller habitats were particularly inhospitable. Our smaller habitats were 30 cm in diameter, and the larger ones were 60 cm. In larger habitats, “unfed” backswimmer and dragonfly nymphs survived well overnight. It is possible that the increased mortality in smaller habitats was due to higher stress associated with conditions specific to smaller habitats which, because of their smaller size might have exhibited faster temperature changes. In the natural environment, these predators are typically found in habitats larger than those used in this experiment [31, 44], thus the reduced space of smaller habitats may have been a factor leading to stress and higher mortality.

In contrast, diving beetles are winged and can maximize their survival by flying to other habitats [45]. Here, habitat size and prey availability strongly influenced the movement of diving beetles. Larger habitats with prey placed 2–4 m away from their initial location were their preferred destinations. Predacious diving beetle species can move in response to food scarcity. In a mesocosm experiment, for example, dispersal responses of the predacious diving beetle species *Laccophilus proximus* were dependent on decreasing prey availability [45]. Prey scarcity in aquatic breeding habitats also caused species of diving beetles *Graphoderus occidentalis* to leave habitats [46]. Therefore, it is likely that diving beetles in this study optimized their foraging efficiencies by searching for prey in nearby habitats as soon as search costs increased due to lower prey density. In this context, their preference for large breeding habitats with prey fits the optimal foraging prediction that, at comparable prey density, larger habitats are preferred as they provide feeding opportunities for a longer period than smaller ones, resulting in fewer flights. The assumption is that diving beetles would seek to minimize the need for flight, which is both risky in terms of predation or not finding another suitable habitat, and energetically costly.

Overall patterns of daily prey consumption from all three predator taxa broadly matched predictions from optimal foraging theory [47]. During the experiments, prey densities were kept equal across small and large habitats resulting in their absolute numbers being double in larger habitats. Being unable to move between habitats, both backswimmer and dragonfly nymph consumption of mosquito larvae depended on the total number of mosquito larvae available and consumed in the larger habitats. Since mosquito larvae were replaced each day, backswimmers and dragonfly nymphs did not consume all the mosquito larvae in a single day; hence, some mosquito larvae remained in the habitats. In contrast, the ability of diving beetles to fly between habitats when prey profitability dropped allowed them to optimize foraging in a different way. Consequently, they were not affected by the lack of prey in some habitats and could move to nearby habitats with higher prey densities. Therefore, habitat size did not impact the number of prey that beetles consumed per day. Overall, the total number of mosquito larvae that the diving beetles consumed did not exceed that consumed by backswimmer and dragonfly nymphs. One potential weakness of this study was that some diving beetles moved to nearby habitats used by other predators which could have biased both their estimates of prey consumption and that of the resident diving beetles, backswimmer or dragonfly nymphs. To assess whether this impacted the study results, analyses were re-conducted after removing all data from artificial habitats where there had been departing or incoming diving beetles. This had no impact on the results of the study.

The size range of predators used in this study broadly matched the most frequent sizes found when sieving aquatic habitats but differed between taxa. Therefore, future studies should focus on establishing prey versus predator size profitability curves in large and small habitats more precisely. This would further benefit from identifying the common predator taxa used to the genus or species level, which is difficult due to lack of taxonomical keys focusing on these regions.

Overall, this experiment highlighted the high predation rates that diving beetles, dragonflies, and backswimmer nymphs can exert on the larvae of *An. arabiensis* and other members of the *An. gambiae* complex. This may explain why *An. gambiae* is frequently only found in smaller temporary habitats where there is often a low diversity of aquatic mosquito macroinvertebrate predators [31]. To provide more insight, semi-field studies could be conducted to assess whether gravid *An. gambiae* females actively avoid ovipositing in habitats where predators are present, as suggested by surveys and small cage oviposition choice experiments [48–50]. The aquatic macroinvertebrate predator taxa used in this study are particularly good candidates for the biocontrol of malaria vectors because both their immature and adult stages feed on mosquitoes.

When given the opportunity, adult dragonflies feed on adult mosquitoes [51]. However, because they are diurnal hunters, they can only prey on *An. gambiae* species at dawn and dusk, when the latter begin flying. While there are still significant challenges in implementing aquatic predator-based malaria mosquito control interventions, including rearing constraints, the foraging and survival data presented here suggest that mosquito larval breeding sites inseminated with backswimmers and dragonfly nymphs would achieve higher suppression levels than those obtained with adult diving beetles. Water boatmen, water striders, and other aquatic hemipterans with predatory larval and adult stages should be treated similarly, as they will also optimize their foraging behaviour differently once they reach adulthood and are able to fly.

## Conclusions

Based on these broad observations, we conclude that treating mosquito habitats with the eggs or young larval stages of insect predators would likely be the best option for effective biocontrol. Consequently, progress in the rearing ecology of these insect taxa is critical. This is such a bottleneck that, in the future, the best aquatic predator biocontrol candidate may well not simply be those that consume the most mosquito larvae per unit time, but rather the taxa and species that can be bred on a larger scale.

## Data Availability

No datasets were generated or analysed during the current study.

## Abbreviations

DDT: Dichlorodiphenyltrichloroethane
WHO: World Health Organization
RBM: Roll Back Malaria
IRS: Indoor residual spraying
ITNs: Insecticide-treated nets
DNA: Deoxyribonucleic acid
(rDNA): Ribosomal deoxyribonucleic acid
PCR: Polymerase chain reactions
dNTPs: Deoxynucleotide triphosphates
MgCl_2_: Magnesium chloride
EDTA: Ethylenediaminetetraacetic acid
SFS: Semi-field system
